# Spontaneous development of Epstein-Barr Virus associated human lymphomas in a prostate cancer xenograft program

**DOI:** 10.1371/journal.pone.0188228

**Published:** 2017-11-16

**Authors:** Alberto J. Taurozzi, Ramprakash Beekharry, Michelle Wantoch, Marie-Christine Labarthe, Hannah F. Walker, Robert I. Seed, Matthew Simms, Greta Rodrigues, James Bradford, Geertje van der Horst, Gabri van der Pluijm, Anne T. Collins

**Affiliations:** 1 Cancer Research Unit, Department of Biology, University of York, York, United Kingdom; 2 Department of Urology, St James University Hospital, Leeds, United Kingdom; 3 Leeds Institute of Cancer & Pathology, University of Leeds, Leeds, United Kingdom; 4 Department of Chemistry, University of York, York, United Kingdom; 5 Department of Urology, Castle Hill Hospital, Cottingham, United Kingdom; 6 Hull -York Medical School, University of York, York, United Kingdom; 7 Department of Pathology, Hull Royal Infirmary, Hull, United Kingdom; 8 Sheffield Institute for Nucleic Acids, Department of Oncology and Metabolism, University of Sheffield, Sheffield, United Kingdom; 9 Department of Urology, Leiden University Medical School, Leiden, The Netherlands; Medizinische Universitat Innsbruck, AUSTRIA

## Abstract

Prostate cancer research is hampered by the lack of *in vivo* preclinical models that accurately reflect patient tumour biology and the clinical heterogeneity of human prostate cancer. To overcome these limitations we propagated and characterised a new collection of patient-derived prostate cancer xenografts. Tumour fragments from 147 unsupervised, surgical prostate samples were implanted subcutaneously into immunodeficient Rag2^-/-^γC^-/-^ mice within 24 hours of surgery. Histologic and molecular characterisation of xenografts was compared with patient characteristics, including androgen-deprivation therapy, and exome sequencing. Xenografts were established from 47 of 147 (32%) implanted primary prostate cancers. Only 14% passaged successfully resulting in 20 stable lines; derived from 20 independent patient samples. Surprisingly, only three of the 20 lines (15%) were confirmed as prostate cancer; one line comprised of mouse stroma, and 16 were verified as human donor-derived lymphoid neoplasms. PCR for Epstein-Barr Virus (EBV) nuclear antigen, together with exome sequencing revealed that the lymphomas were exclusively EBV-associated. Genomic analysis determined that 14 of the 16 EBV^+^ lines had unique monoclonal or oligoclonal immunoglobulin heavy chain gene rearrangements, confirming their B-cell origin. We conclude that the generation of xenografts from tumour fragments can commonly result in B-cell lymphoma from patients carrying latent EBV. We recommend routine screening, of primary outgrowths, for latent EBV to avoid this phenomenon.

## Introduction

The limitations of current preclinical models are increasingly cited as a key cause of the low success rate of oncology drug development [1]. Traditionally, preclinical models of prostate cancer are cell lines cultivated in monolayer or xenografts derived from them. Unlike other solid tumours, few prostate cell lines are available and as such do not represent the heterogeneity and complexity of this disease. Indeed, preclinical efficacy of anticancer agents has rarely translated into clinical efficacy [2]. A key consideration is the length of time these cell lines have been in culture, undergoing extensive adaptation and selection. Patient-derived xenograft (PDX) models, based on direct implantation of fresh cancer tissue specimens into immunodeficient mice have become reliable models for preclinical research in many types of cancer [3]. PDXs are reported better predictors of response and retain the cellular heterogeneity, architecture, and molecular characteristics of the original cancer [4], offering the possibility of individualized cancer treatment, guided by molecular profiling of the PDX. In contrast to other tumour types, prostate cancer xenografts are notoriously difficult to establish [5]. The reasons for that are complex and are most likely due to poor sampling of the tumour, the strain of mouse, engraftment site and cell rather than tissue engraftment. Some investigators have successfully generated xenografts from purified populations of cells from human cancers [6–8], using mouse strains deficient in both innate and adaptive immunity [9], but the majority generate xenografts from tissue fragments [10]. Supplementing mice with androgens and the use of mouse embryonic mesenchyme has improved engraftment efficiency [11], but the biggest improvement has been the development of mice lacking natural killer (NK) T cells, particularly for tumours that are particularly difficult to establish as a xenograft [10]. Despite these improvements, there are very few prostate cancer PDX lines available that are ‘near-patient’ and from the primary disease [12].

The goal of this study was to generate a panel of prostate cancer xenografts as preclinical models for drug screening and biomarker development. To improve efficiency we implanted tumour fragments into the immunocompromised Rag2^-/-^γC^-/-^ mouse. Here we report on the characterisation of a panel of 20 stable PDXs lines. After careful validation we established that only 15% (3/20) were typical of prostate cancer.

## Material and methods

### Generation of xenografts

All animal work was approved by the University of York Animal Procedures and Ethics Committee and performed under a United Kingdom Home Office License (POB5AE607). *Rag 2*^*tm1*.*1Flv*^ *Il2rg* ^*tm1*.*Flv*^ also known as Rag2^-/-^ γc^-/-^ mice were bred in the Biology Service facility (BSF), Department of Biology, University of York. The mice used for xenografts were between 6–8 weeks old.

Human prostatic tissue was obtained from 147 adult patients undergoing radical prostatectomy and trans-urethral resection (TUR) for prostate cancer, with informed written consent (NHS Research Ethics Approval (REC) 07/H1304/121). Some patients had undergone androgen-deprivation therapy prior to TUR. The specimens were sectioned and examined by pathologists for histological analysis subsequent to xenografting. Tissue pieces were grafted subcutaneously into recipient Rag2^-/-^γC^-/-^ mice. Biopsies from hormone naïve patients, mice were engrafted with 90 day hormone release pellets (12.5mg of 5αdihydrotestosterone; DHT) at the time of tissue implantation. Mice were bred in our facility, and were housed in individually ventilated cages. Once tumours reached 1.5cm^3^ (considered a humane endpoint) the mice were sacrificed (by cervical dislocation) and the tumours were either re-implanted (under anaesthesia) into further mice or the tissue was processed for further experiments. To maintain the tumour xenograft as ‘near-patient,’ tumours were re-established from frozen cells after 5 passages in mice. Single cells were isolated from xenograft tumours as previously described [13]. However, in this study mouse cells were further depleted using the Mouse Cell Depletion kit (Miltenyi Biotec; cat # 130-104-694) with 98±2% purity.

### Histologic evaluation of xenografts

Transplanted tumours were fixed in 10% neutral buffered formalin, paraffin-embedded, and haematoxylin-eosin (H&E) stained as described previously [13]. Primary outgrowths were analysed by immunohistochemistry for expression of clinically-relevant biomarkers (androgen receptor (AR); polyclonal N-20 (Santa Cruz Biotechnology) & clone 441 (ThermoFisher Scientific), prostate specific antigen (PSA) clone 28/A4; Abcam, human pan cytokeratin (clones C-11, PCK-26, CY-90, KS-A13, M20, A53-B/A2; Sigma-Aldrich) and chromogranin A (clone LK2H10; Invitrogen). Prostate tissue, from patients with BPH and cancer, was used as a positive control for each antibody tested. Non-specific binding was assessed using isotype controls and secondary only antibodies.

### Flow cytometry

Cells harvested from xenografts were analysed for the expression of human CD44 (clone DB105; Miltenyi Biotec), human CD24 (clone 32D12; Miltenyi Biotec), human EpCAM (clone CD326; Miltenyi Biotech), human B lymphocyte antigen, CD19 (clone HIB19; eBioscience), human neural cell adhesion molecule, CD56 (clone AF12-7H3; Miltenyi Biotec) and human CD45 (clones H130 and 2D1; eBioscience) following mouse cell depletion (Miltenyi Biotech).

All cells were analysed on a Cyan ADP flow cytometer (Dako Cytomation) and data processed using Summit v4.3 software (Beckman Coulter). Based on flow cytometric analysis we estimated >98% of cells were donor-derived.

### Short tandem repeat (STR) profiling

Xenografts were validated as unique to the patient donor by short tandem repeat (STR) DNA fingerprinting using the Promega Powerplex 16 system, according to the manufacturer’s instructions (Promega). The STR profiles of all xenografts were matched to their respective lymphocyte DNA.

### Androgen deprivation therapy

Androgen ablation was carried out on intact mice, supplemented with 12.5mg 5α- DHT; Innovative Research of America). 90 day release pellets were sutured in place 2 weeks before inoculation of cells. In vivo efficacy was determined in mice carrying serially-transplantable human tumour xenografts. Single cells were generated from xenografts, as previously described, and Rag2^-/-^γC^-/-^ mice were inoculated with 10^4^–10^5^ tumour cells. Once tumours reached approximately 500 mm^3^ in volume, the 5αDHT pellet was replaced with those continuing either Flutamide or placebo, and mice were randomized to treatment and control arms (http://www.randomization.com) for blinded assessment of tumour volume.

Tumour volume was evaluated twice per week by caliper measurement using the formula; tumour volume = (length x width^2^)/2. Relative tumour growth inhibition/regression was calculated as follows: T/C = (T_i_-T_0_/C_i_-C_0_). T_i_ and C_i_ represent tumour size, of treatment and control group respectively, at the end of the experiment; T_0_ and C_0_ represent tumour size at initiation of experiment. Tumour response was also calculated using a rate-based T/C measurement which uses all the data and is based on the ratio of the fitted growth rates. Power analysis was used to calculate a sample size of 8 animals per group (with 90% power and a significance level of 5%).

### Cell lines

The cell lines used in this study were obtained from the European Collection of Authenticated Cell Cultures (ECACC). 22RV1 (Cat. # 05092802), LNCaP (Cat. # 89110211) and VCap (Cat. # 06020201) human prostate cancer cell lines were cultured in RPMI 1640 (Gibco) containing 2mM L-Glutamine and 10% foetal calf serum (FCS). The AR negative prostate cancer cell line (PC3; Cat. # 90112714) was cultured in Ham’s F12 medium (Lonza) containing 2mM L-Glutamine and 7% FCS.

### Quantitative RT-PCR

Total RNA was extracted from mouse-depleted xenografts using Qiagen RNeasy mini-columns, according to the manufacturer’s protocol. RNA was reverse transcribed, using random hexamers (Invitrogen) and reverse transcriptase (Superscript II, Invitrogen). Real time PCR was carried out using SSoFast EvaGreen Supamix (Biorad). Reactions were prepared following manufacturer’s protocols. All reactions were carried out in triplicate on 96-well PCR plates in a CFX96 real time PCR detection system and data analysis was performed using CFX manager software (Bio-Rad). The following primer sets were used; flAR: 5′- CCAGCTTGCTGGGAGAGCGG-3′and 5’- CTGGCGTGGTGCGTCCCTTC- 3’, AR-V1: 5′- CCATCTTGTCGTCTTCGGAAATGTTATGAAGC-3′ and 5′-CTGTTGTGGATGAGCAGCTGAGAGTCT-3′ and 5′- TTTCTTCAGTCCCATTGGTG-3′, AR-V7: 5′- CCATCTTGTCGTCTTCGGAAATGTTATGAAGC-3′ and 5′-TTTGAATGAGGCAAGTCAGCCTTTCT-3′, GAPDH: 5′-GGACACGGAAGGCCATGCCA-3′ and 5′- AAGGTGAAGGTCGGAGTCAA-3′. 22RV1 cell cDNA was used to create a standard curve. Relative expression was evaluated using the relative standard curve method; normalizing to GAPDH and a calibrator (LNCaP or PC3 cell line). AR variant primers were obtained from Donald J Tindall, (Mayo Clinic, Minnesota, USA). Each sample was run in triplicate.

### Whole exome sequencing and data analysis

Genomic DNA was extracted using Qiagen’s DNeasy Blood and Tissue kit from patient lymphocytes and mouse cell-depleted xenograft tumour cells. Whole exome sequencing was performed by Eurofins Genomics. Sequencing of libraries was performed on a HiSeq2500 (Illumina).

#### Mutation calling

Read pairs were mapped against the human genome (build 38) using BWA “mem” algorithm with default parameters [14]. The resulting bam files were then pre-processed in preparation for somatic mutation detection using the Genome Analysis Toolkit (GATK) v3.5 best practice pipeline [15] and dbsnp version 144 in the base recalibration step [16]. MuTect v1.1.7 was then applied to compare the resulting bam files from tumour and matched donor lymphocytes to call somatic mutations [17]. Mutations were annotated using the Ensembl Variant Effect Predictor [18] and non-silent protein coding mutations taken forward for further consideration. The somatic status of each SNV and their prevalence in clinical prostate cancer samples was assessed using the COSMIC database [19].

#### Copy number profiling

As for the mutation calling, read pairs were mapped against the human genome (build 38) using BWA “mem” algorithm with default parameters [14]. Duplicate reads were removed, as were reads achieving mapping quality below 37. Depth of coverage at each position targeted by the Nextera Exome capture kit was calculated using GATK “DepthOfCoverage” tool [15] and the resulting tumour and normal profiles input to ExomeCNV R package using default parameters [20]. Gene level log2 copy number ratios were then parsed using custom Perl scripts, with those achieving |log2 ratio| > 0.50 taken forward for further consideration.

### Quantitative PCR

Genomic DNA was extracted using Qiagen’s DNeasy Blood and Tissue kit from patient mouse cell-depleted xenograft tumour cells. EBV was detected by qPCR using the following primers; EBNA-1 fwd AGATGACCCAGGAGAAGGCCCAAGC and EBNA-1 rev CAAAGGGGAGACGACTCAATGGTGT. The EBV copy number per cell was calculated by normalising the Cq to the single copy gene GAPDH which was assessed using the following primers; GAPDH fwd ATGCTGCATTCGCCCTCTTA and GAPDH rev GCGCCCAATACGACCAAATC. The assay utilised SsoFast EvaGreen Supermix (Bio-Rad) primers at a final concentration of 400nM and 10ng of input DNA. Samples were analysed on FrameStar ® 96 well plates (4titude) using the CFX96 qPCR system (Bio-Rad) and data analysis was performed using CFX manager 2.0 software (Bio-Rad). Amplified products were identified on a 1.5% Agarose TBE gel.

To determine androgen receptor (AR) copy number the following primers were used:

AR fwd TCATTATCAGGTCTATCAACTCTT and AR rev GTCATCCCTGCTTCATAACATTTC and Dystrophin (DMD) fwd TTGGTTGCCAGTTATGGGCT, DMD rev CCAGCTGTCATGCAAAACCC and GAPDH. The AR copy number per cell was calculated by normalizing the Cq of the AR to that of GAPDH and DMD. DMD is located on the X-chromosome and was used to distinguish between AR amplification and copy number alterations. Controls included female DNA, donor lymphocytes and the cancer cell line VCaP, which has an amplified AR gene.

### IgH gene rearrangement assay

Clonality was evaluated by PCR for V-J gene rearrangements of the IgH gene using the IdentiClone™ diagnostic kit from Invivoscribe. This kit has been validated for use in the diagnosis of patients with suspected lymphoproliferation. The assay employs multiple consensus DNA primers that target conserved genetic regions within the IgH gene. The test includes 6 master mixes targeting the conserved framework (FR) of the variable (V) regions and the conserved joining (J) regions, as well as the diversity (D) and joining regions. DNA bands were either visualized on a non-denaturing 6% polyacrylamide TBE gel, or for downstream Sanger sequencing, PCR products were loaded onto a 1.5% Agarose TBE gel.

### Blue-white screening

Amplicons generated from the IgH assay were extracted from 1.5% agarose gels and purified using the QIAquick Gel Extraction Kit according to the manufacturer’s instructions (Qiagen). Products were ligated into the pGEM®-T Easy vector using the pGEM®-T Easy system (Promega), transformed into JM109 High Efficiency Competent Cells (Promega UK) and subsequently plated onto LB/carbenicillin/IPTG/X-Gal plates. Following amplification of individual colonies plasmid DNA was extracted, checked for the presence of insert by restriction digestion with EcoRI (Promega) and sequenced using Sanger sequencing (Applied Biosystems 3130XL) with M13-47 (CGCCAGGGTTTTCCCAGTCACGAC) and M13 rev -26 (GGAAACAGCTATGACCATG) primers.

### T-cell receptor gamma chain gene rearrangement assay

Clonality was evaluated by PCR for V-J gene rearrangements of the T cell receptor gamma gene (TCRG) using the IdentiClone™ TCRG gene kit from Invivoscribe. This kit has been validated for use in the diagnosis of patients with suspected lymphoproliferation. The assay employs multiple consensus DNA primers that target conserved genetic regions within the TCRG gene. The test includes 3 master mixes targeting the conserved flanking regions around the V-J rearrangement. DNA bands were visualized on a non-denaturing 6% polyacrylamide TBE gel.

### Statistical analysis

Associations between tumour characteristics and various clinical parameters (Gleason grade, tumour stage, hormone status, PSA status) were investigated using Fisher’s exact test. Repeated Measures Parameter Analysis (InVivoStat) was used to assess tumour growth over time. Pairwise tests were carried out to assess the difference between predicted means. *P* <0.05 was considered significant for all statistical analysis.

## Results

### Establishment of xenografts from tumour fragments

Primary tumour fragments, from 147 patients, were implanted subcutaneously into Rag2^-/-^γC^-/-^ mice resulting in tumour outgrowths from 47 biopsies (32% primary outgrowth rate). Seventeen patients (17 of 47 or 36%) from this cohort had not received any form of therapy whereas the remaining 30 patients (64%) had received hormone therapy alone or hormone treatment and radio/chemo therapy (15%). From 20 patients, we established and expanded 20 transplantable tumour lines for a minimum of 3 generations (14% take rate). Of those, seven patients had not received any form of therapy, 10 had received hormone therapy alone and 3 had received hormone therapy and radio/chemotherapy (Table 1).

**Table 1:** Characteristics of xenografts and corresponding donor tumours.

Sample ID	Patient information			Xenograft information							
	Age	Pathology	Hormone Status	^a^Latency (mo)	^b^Doubling time (d)	AR	Androgen sensitivity	^c^flAR	AR Variants	^d^X aneuploidy	AR amplification
Y042	56	G3+4, T2	HN	4	4	+	Partial	+	+		
H016	67	G4+5, T3a	HN	3	9	-	No	+	+		
H024	69	G4+3, T3b	HN	5	9	-		+	+		
H042	63	G3+4, T2c	HN	4	22	-		-	+		
H084	61	G4+3, T3a	HN	7	25	+	No				
H082	53	G3+4, T3a	HN	6	40	+	No	+	+		
H087	68	G3+4	HN	4	14	+					
H050	60	G3+4, T2c, b1	HR	6	21	-		-	-		
H288	79	G4+3, T2c, m1	HR	4	9	-					
H070	70	G3+4, T2c	HR	4	16	-					
H027	68	G5+4, n1	HR	6	25	+					
H107	71	G4+4, T2b	HR	5	17	+					
H427	69	G4+5, T3b, n1, b1	HR	18	55	+				+	+
H460	76	T3	HR	7	14					-	-
H493	68	G4+5, T3b, n1, b1	HR	4	20					-	-
Y019	70	G4+5	CRPC	5	7	-		+	+	-	-
Y018	75	HC	CRPC	6	27	+				-	-
Y056	67	G5+4	CRPC	4	18	+		+	+		
H149	78	G4+5, T4, n1, m1	CRPC	12	15	+					
H455	67	m1	CRPC	10	28	+				+	+

2mm core biopsies, from men undergoing radical prostatectomy or trans-urethral resection for prostate cancer, were engrafted, subcutaneously, into Rag2^-/-^γc^-/-^ mice. All xenografts were derived from primary prostate cancer. Gleason score and stage, at biopsy are shown. HC = Hormone changes following ADT. HN = Hormone naïve. HR = Donor hormone responsive at time of biopsy. CRPC = castrate-resistant prostate cancer.

^a^time taken for primary outgrowth until establishment of a transplantable tumour line.

^b^tumour diameter doubling time of stable PDXs.

^c^expression of flAR, ARV1 or ARV7 by qRT-PCR.

^d^X aneuploidy and AR amplification were determined by qPCR.

AR expression status refers to protein expression unless otherwise stated.

To evaluate which clinical characteristics correlated with tumour take the primary and stable xenograft outgrowth rates were compared across each clinical characteristic using Fisher’s exact test. Stable xenograft development was most likely from patients who had undergone hormone treatment (OR: 2.9, 95% CI: 1.1–8.1, *P* = 0.04), with only Gleason grade 7 and above yielding stably transplantable xenografts.

PDX tissue was genotyped at primary outgrowth and at alternate generations using STR profiling. A comparison was made with lymphocytes from the patient donor (S1 Table). Complete concordance was observed for 15 models at all 16 loci. Chromosomal loss or deletions was observed in models Y042, H427, Y019 & H455, with gains observed in model Y056. Whilst all PDX tissues were confirmed to be patient-derived at the first generation, xenograft H070 was confirmed as exclusively murine at the fourth generation, and was excluded from the study.

Latency (time from initial engraftment until establishment of a transplantable tumour line) ranged from 3 to 18 months (median, 5 months, Table 1). Latency was not associated with doubling time or donor pathology as tumour lines with the shortest latencies (3–4 months) had doubling times which ranged from 4–22 days and were derived from hormone naive and CRPC patients (Table 1).

#### Xenograft histopathology and molecular characteristics

**Fig 1:**
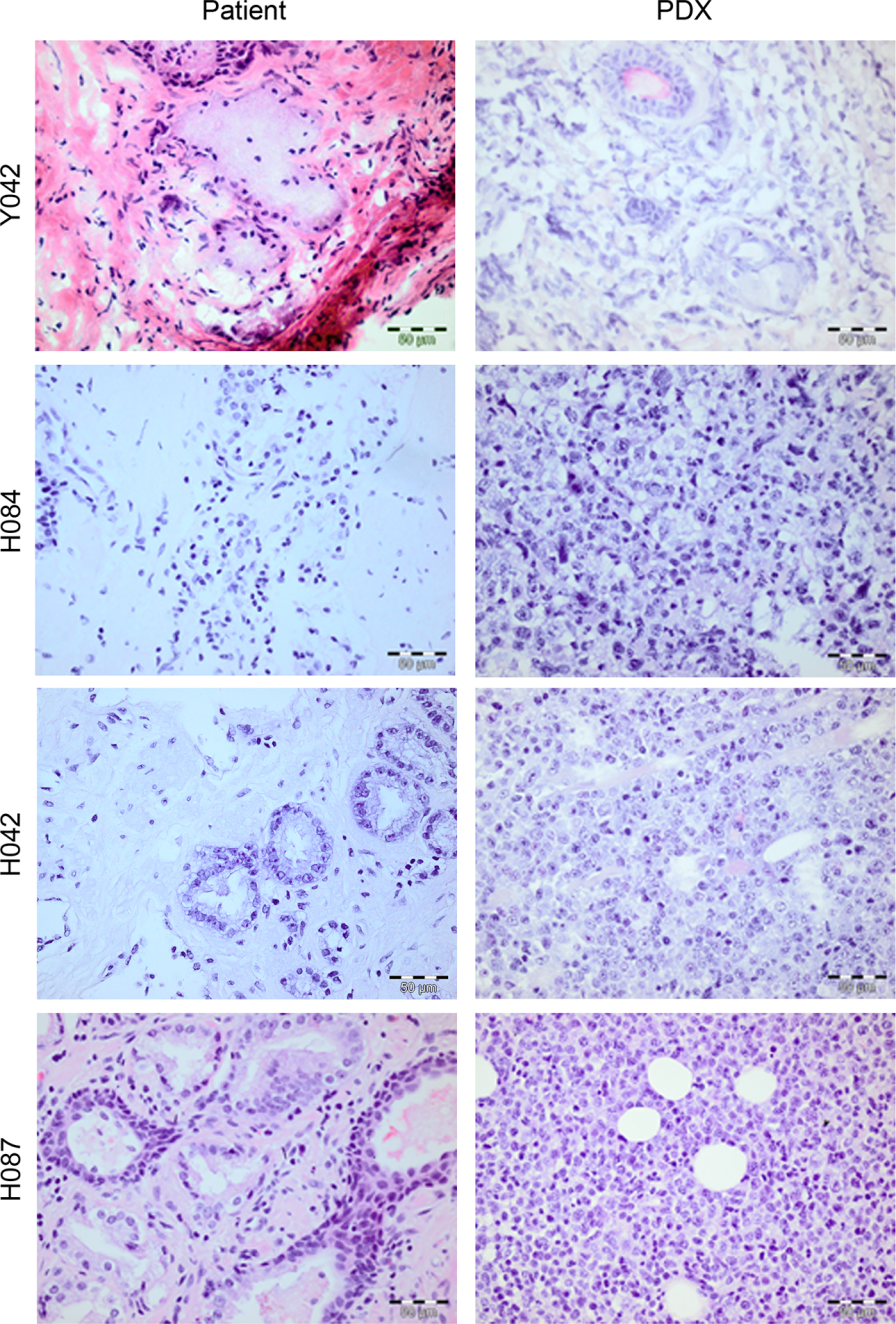
Primary tumour xenografts derived from treatment-naïve human prostate tissue specimens. H&E sections of representative prostate cancer xenografts and their corresponding human donor sample. Note loss of glandular architecture.

**Fig 2:**
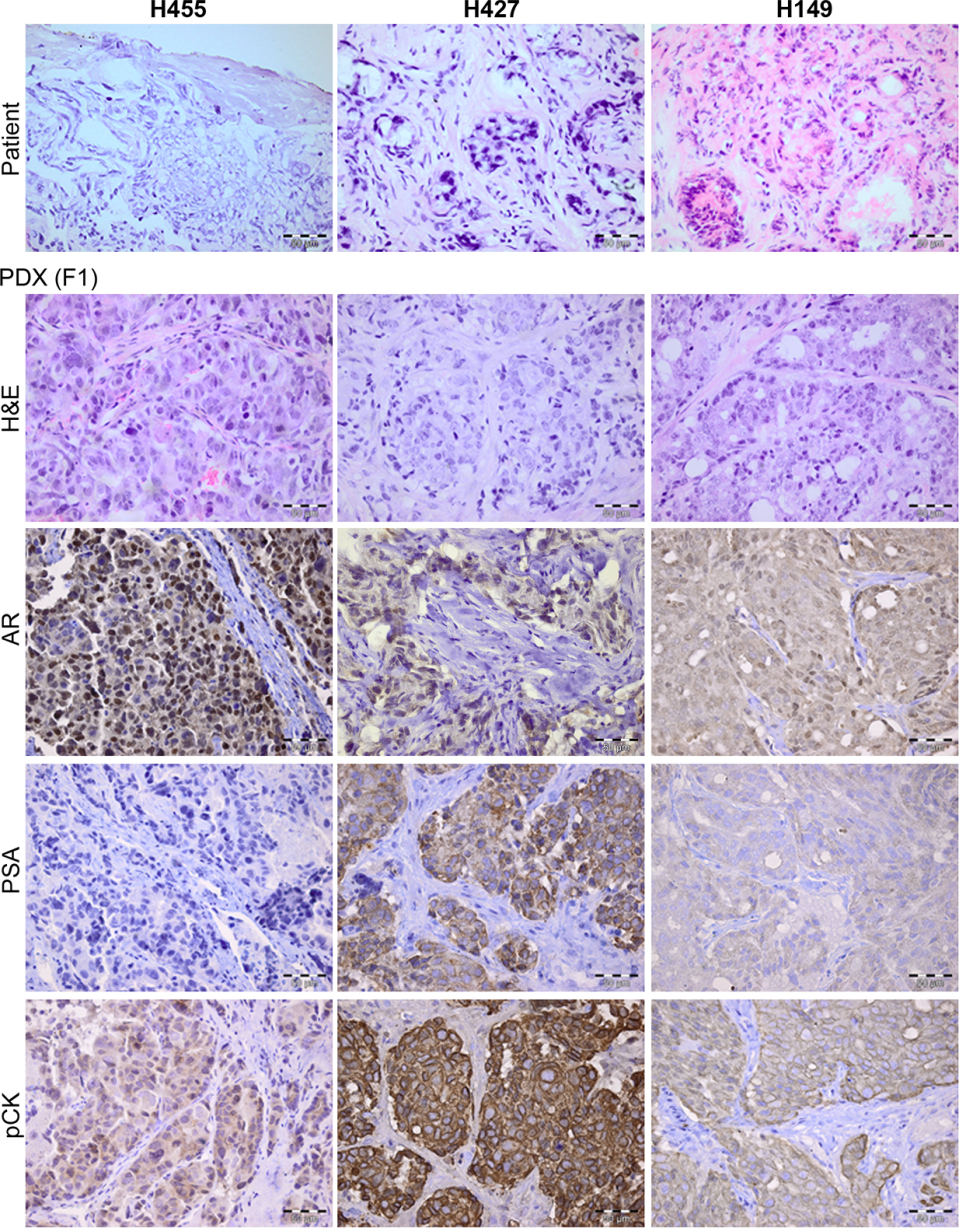
Primary tumour xenografts derived from human prostate tissue specimens demonstrating typical features of prostate adenocarcinoma. H&E sections of human donor sample (upper panel) and corresponding xenograft (lower panel). Xenograft tissue sections were stained with antibodies raised against human AR (clone 441 at 1:50), PSA (1:25) and pan-cytokeratin (1:800). Human tissue, from patients with BPH or cancer was used as a positive control. Non-specific signal was assessed using isotype controls and secondary only antibodies. Xenograft images are from primary outgrowths (annotated as F1). Magnification x400.

We next evaluated whether stable xenografts retained histologic features and biomarker expression patterns consistent with prostate cancer. The diagnosis of prostate cancer and degree of tumour differentiation was assessed by a uropathologist. The majority of stable lines were derived from patients who had undergone androgen ablation making the assessment of these specimens difficult, due to hormonal changes. Nonetheless, the xenografts derived from hormone naïve patients did not resemble their matched patient tumour, specifically in the degree of differentiation (Fig 1,S1 Fig). All had features of poorly differentiated carcinoma, with nuclear atypia, high nuclear-to-cytoplasmic ratios and loss of glandular architecture. Only three xenograft lines matched their patient donor in the degree of differentiation, and expression of biomarkers typical of prostate adenocarcinoma (Fig 2, S1 Fig). Whilst the remaining seventeen lines were largely devoid of cytokeratin and PSA expression, we observed expression of the flAR at the protein and RNA level as well as expression of AR variants. (Table 1, S1 Fig, S2 Fig). Furthermore, we observed a partial response to flutamide in 1 of 4 xenografts (Y042) and a small, but significant increase in the rate of growth in the flutamide arm, in mice bearing H084 tumours (S2 Fig).

**Fig 3:**
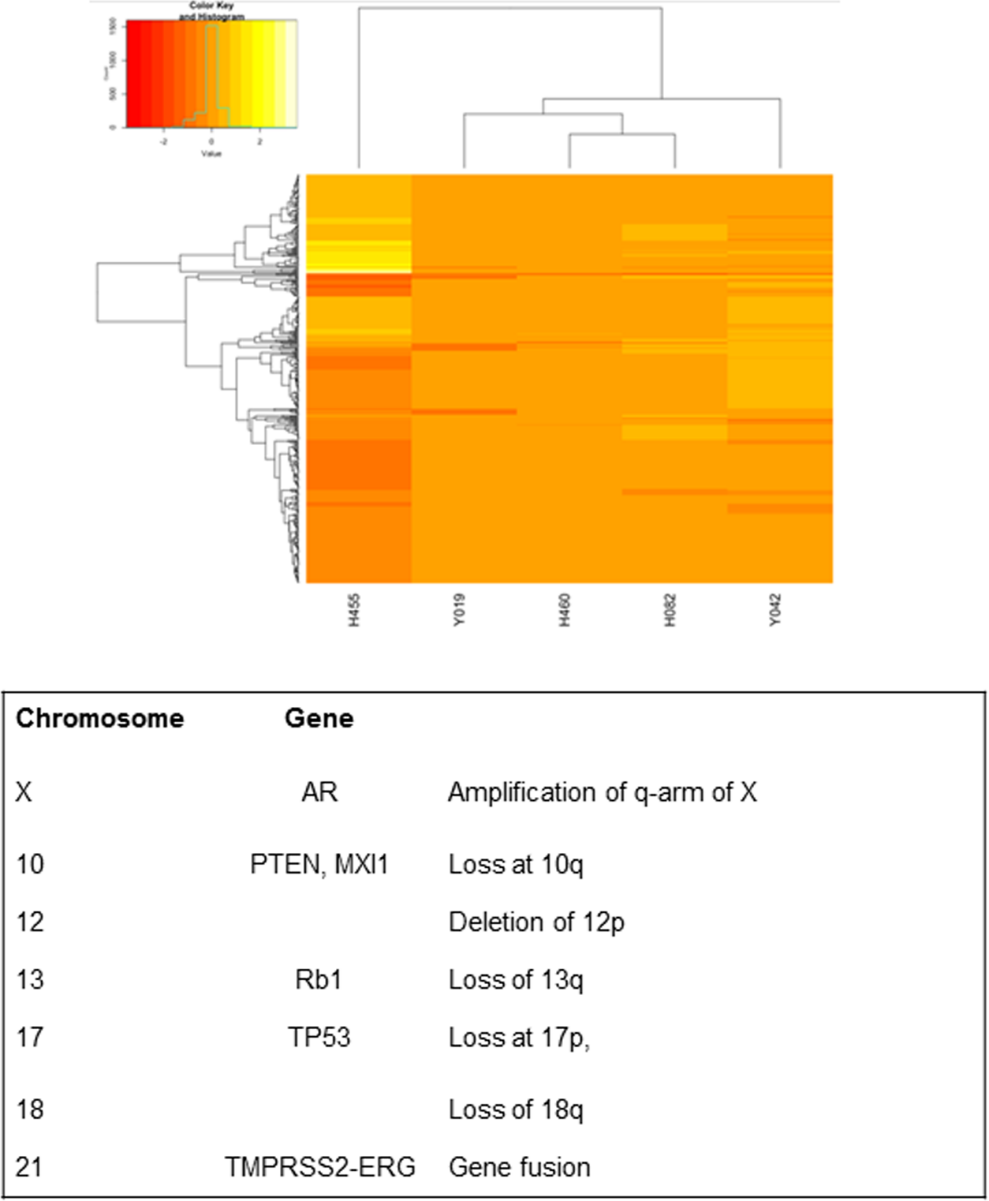
Unsupervised hierarchical clustering of copy number segmentation profiles. PDX, H455 is as an outlier and the most aberrant in terms of copy number. Table listing CNVs found in PDX H455.

Genome instability is implicated in the development and progression of prostate cancer and is a feature of many cancers [21, 22]. Xenografts were compared with their matching lymphocytes using whole exome sequencing to identify copy number aberrations and cancer gene mutations. We were unable to perform a comparative analysis of the original tumours versus xenograft due to limiting amounts of patient tumour tissue. To avoid confounding signals, xenografts with greater than 1% mouse component were excluded from the analysis. There was little evidence for the presence of common prostate cancer SNVs (single nucleotide variants) in the PDX tumours other than TP53 (H455) (S2 Table). However, unsupervised hierarchical clustering of copy number segmentation profiles clearly showed H455 as an outlier and the most aberrant in terms of copy number (Fig 3, S2 Table). Similar to previously published prostate cancer studies [23], recurrent chromosomal abnormalities included losses on chromosomes 10q (including PTEN and MXI1), 12p, 13q (including Rb1), 17p (including TP53), 18q, 6q and 9q (Fig 3, S2 Table). Significantly, the 3Mb deletion between ERG and TMPRSS2 on Ch21 was indicative of the TMPRSS2-ERG fusion product, a major molecular hallmark of prostate cancer [21]. Gains included the q-arm of X, which was confirmed by PCR for AR (Table 1). In contrast, no obvious prostate cancer associated changes were found for the remaining xenografts, which together with the lack of prostate cancer markers and response to castration prompted further investigation.

### Epstein-Barr virus and lymphoma development in prostate cancer xenografts

**Fig 4:**
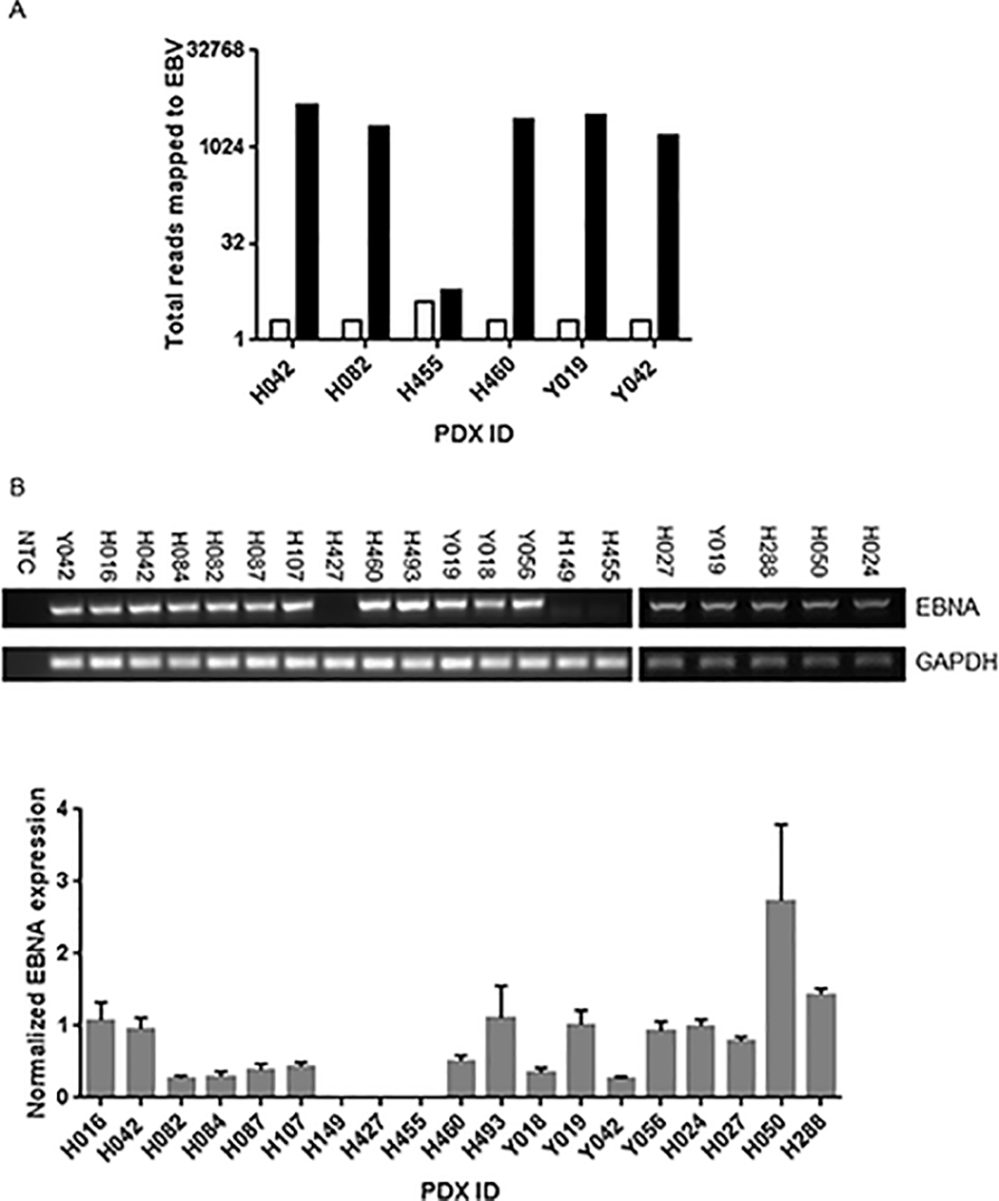
EBV status of PDX lines. **A.** Total reads, generated from whole genome exome sequencing, mapped to EBV. Donor Lymphocytes (open bars) were compared to PDX lines (solid bars). **B**. PCR amplification of EBNA and GAPDH in PDX lines (upper panel). NTC = non template control. Lower panel; normalized EBNA expression in PDX lines.

In 2015, Wetterauer and colleagues [24] published findings describing the development of human lymphomas in a prostate cancer xenograft program. Given the importance of EBV in the pathogenesis of lymphoproliferative disorders in immunocompromised humans we firstly evaluated the xenografts for the presence of EBV DNA. We interrogated the sequences generated from exome sequencing and compared xenografts to donor lymphocytes (Fig 4A). The total reads mapped to EBV were significantly higher in five of six xenografts compared to their matched donor lymphocytes which we calculated as equivalent to 1–2 copies of the EBV genome. In a larger series, we determined the presence of latent EBV infection by qPCR for EBV nuclear antigen (EBNA); which is found in all EBV-related malignancies and is critical for the replication of the episomal EBV genome [25]. As shown in Fig 4B, 16 of 19 (84%) PDX were positive for latent EBV. We could exclude cross contamination between xenografts as the source of EBV infection as STR profiling confirmed that the xenografts are genetically distinct and identical to the patient donor (S1 Table).

**Fig 5:**
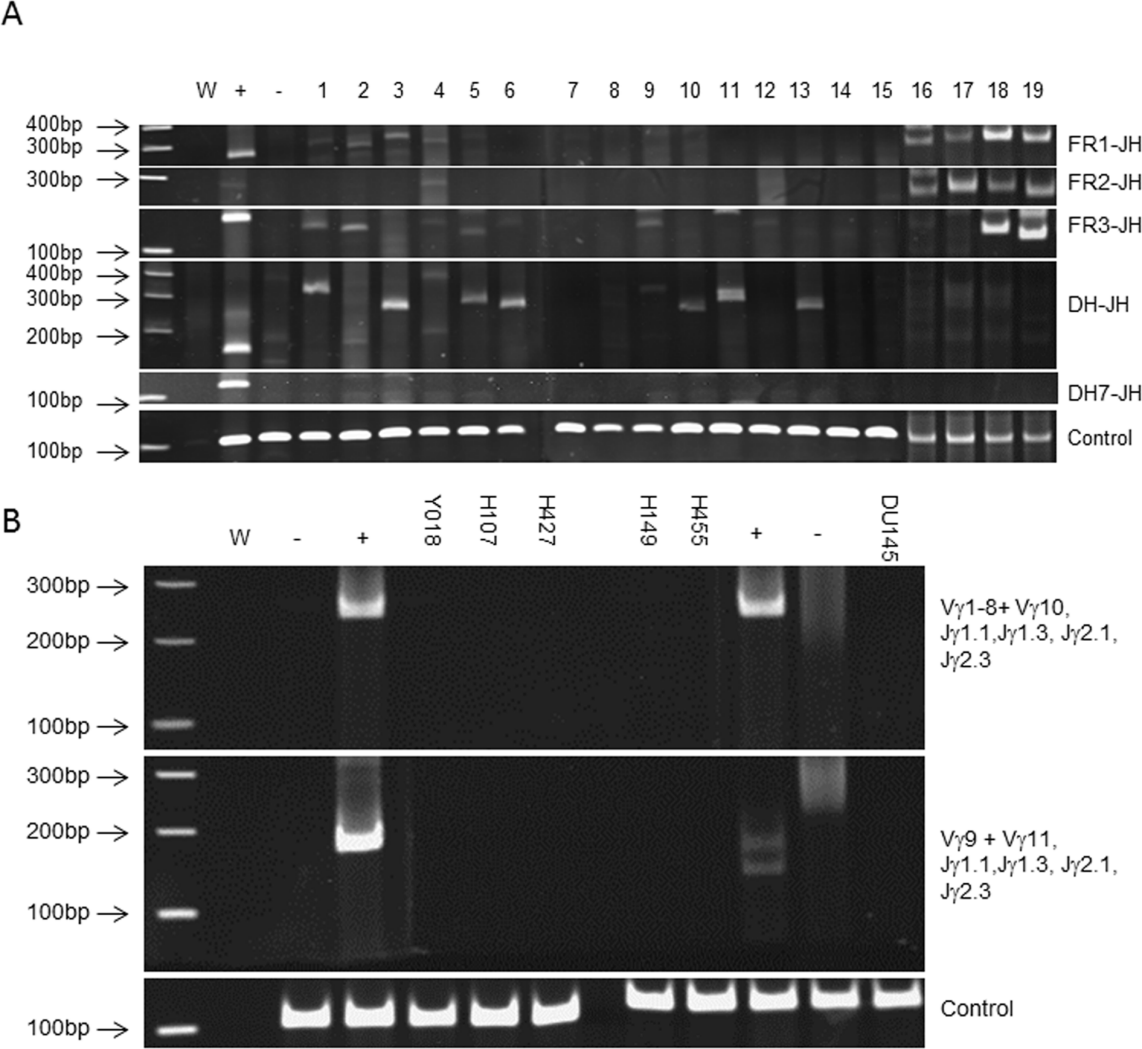
IGH and T cell receptor gamma chain gene rearrangements in a PDX panel. **A**. PCR amplification of IGH gene VJD regions using a multiplexed PCR. W = water control. + = B cell lymphoma clonal control.— = negative control (prostate epithelial primary culture). Targets, FR1-JH: Y042 (L1) H016 (L2), H042 (L3), H084 (L4), H027 (L16), H288 (L17), H050 (L18), H024 (L19) are within the valid size range (310-360bp). FR2-JH: H084 (L4), H027 (L16), H288 (L17), H050 (L18), H024 (L19) are within the valid size range (250-295bp). FR3-JH: Y042 (L1), H016 (L2), H084 (L4), H082 (L5), H087 (L6), H460 (L9), Y018 (L12), H050 (L18), H024 (L19) are within the valid size range (100-170bp). DH-JH: H084 (L4), H087 (L6), H493 (L10), Y019 (L11), Y056 (L13) are within the valid size range (110–290 and 390-420bp). DH7-JH: valid size range is 100-130bp **B**. PCR amplification of TCRG V-J regions using a multiplexed PCR. W = water control. + = Positive controls (lanes 4 & 10; T cell lymphoma clonal controls),— = negative controls (lane 3; prostate primary culture, lane 11; polyclonal control, lane 12; DU145 prostate cell line). Targets, Vγ1–8+ Vγ10, Jγ1.1,Jγ1.3, Jγ2.1, Jγ2.3: Valid size range 145–255 bp. Vγ9+ Vγ11, Jγ1.1,Jγ1.3, Jγ2.1, Jγ2.3: Valid size range 80–220 bp.

To confirm that the source of latent EBV was due to the proliferation of human B lymphocytes we looked for immunoglobulin heavy chain (IgH) rearrangements which occur specifically in B lymphocytes during maturation (Fig 5A). Using a clinical diagnostic kit which utilises a multiplex PCR targeting the VJD regions of the IgH gene we concluded that 15 of 16 EBV^+^ xenografts arose from a single B cell clone. We also verified that 3 EBV—xenografts (H427, H455, H149) had not arisen from human B cells. To confirm these findings, in particular where there was some ambiguity (e.g. band was not prominent or just outside the valid size range) we cloned and sequenced a number of amplicons (S3 Table). A subsequent BLAST search showed that xenograft Y018 had not arisen from a B cell, despite its EBV status.

**Fig 6:**
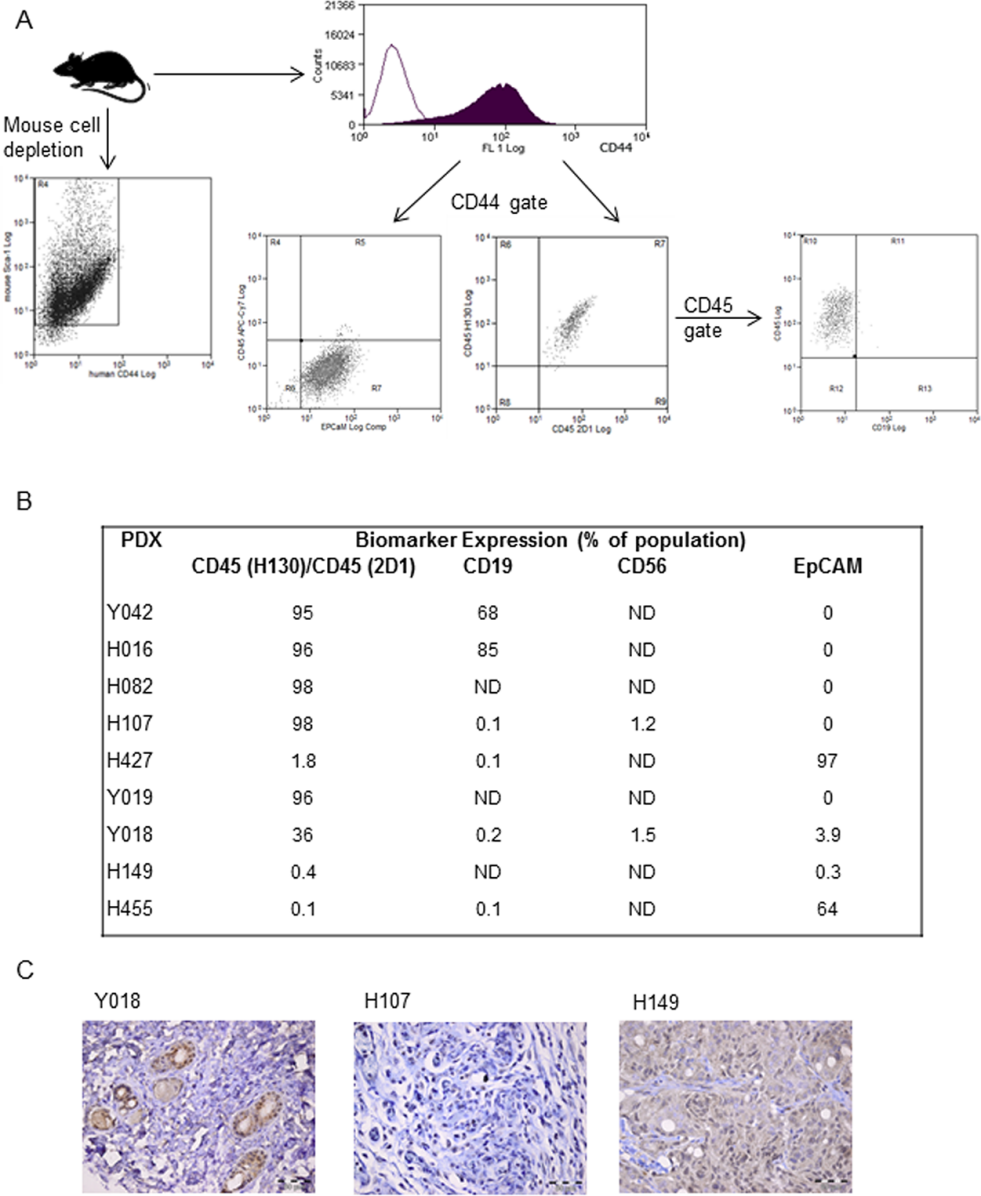
Flow cytometric gating strategy and analysis of PDX panel for epithelial and haematopoietic lineage markers. **A**. Serially transplantable PDX tumour were depleted of mouse cells before labelling with human specific antibodies to CD44, CD45, CD19, CD56 and EPCaM. **B**. Table of percentage of cells expressing specific markers. LNCaP was used as a positive control for EPCaM expression and PC3 was used as a negative control for CD45, CD19 and CD56 expression. **C**. IHC of Chromogranin A expression in PDX lines.

To exclude the possibility that the two remaining EBV^+^ xenografts (Y018, H149) arose from clonal T cell populations, we tested for T-cell receptor gamma chain gene rearrangements (which occur during T cell maturation) using a multiplex PCR targeting several V regions within the gene locus. We established that the remaining PDXs had not arisen from a T cell clone (Fig 5B). Further phenotypic analysis, using a combination of flow cytometry and IHC, established that H107 and Y018 were unlikely to have arisen from NK cells, due to lack of reactivity for CD56 (Fig 6B). Nevertheless, we concluded that H107 is more typically lymphoblastic (CD45^+^/EpCAM^-^/ChrA^-^) whereas Y018, whilst not expressing EpCAM does express the neuroendocrine marker Chromogranin A (Fig 6C). These analyses also confirmed that the EBV^-^ PDX were typically prostate cancer with some neuroendocrine features; such as Chromogranin A expression, observed in xenograft H149 (Fig 6C).

## Discussion

In the present study we aimed to generate human prostate cancer xenograft models to assess their feasibility as preclinical models for drug screening and biomarker development. After careful validation we established that only 2% of the biopsies engrafted resulted in a stable line, resembling prostate cancer. The remaining stable xenografts were classified as donor-derived lymphoma, associated with EBV.

Histologically, the xenografts did not resemble their matched donor tumour, particularly from Gleason 7 disease. Our initial assessment of poorly differentiated adenocarcinoma was based on reactivity for markers associated with prostate cancer. Chen and colleagues similarly described atypical undifferentiated morphology from a series of xenografts generated in NSG mice [26]. They reported sporadic reactivity for AR, cytokeratin and EpCAM and suggested rare, undifferentiated clones from the donor tumour had established the xenograft. In support of these data is the finding that establishment of human leukaemia in immunodeficient mice selects and expands a more aggressive malignancy, recapitulating the process of relapse in patients. Comparisons of paired diagnosis and relapsed samples showed that with regard to genetic lesions, xenograft leukaemias more closely resembled relapse samples than bulk diagnosis samples [27]. The partial response to flutamide and the presence of AR variants further confounded the provenance of the xenografts. Whilst it has been reported that up to 70% of B cell lymphomas express AR [28], we are not aware of any publications reporting expression of AR variants in human lymphoma.

Our finding that there was little evidence for the presence of common prostate cancer SNVs in the atypical prostate cancer xenografts prompted us to investigate further. We screened for the presence of EBV because of its link with the pathogenesis of lymphoproliferative disorders. EBV is a human herpesvirus that infects over 90% of humans persisting for the lifetime of immunocompetent individuals as an asymptomatic, latent infection of the B-lymphocyte pool. However, immunocompromised individuals, such as those receiving immunosuppressive drugs are at risk of developing B-cell lymphomas [29]. The presence of EBV could not be confirmed in donor specimens due to the limited supply of tissue and we reasoned that because of the efficiency of the immune system in suppressing EBV, in immunocompetent individuals, we would not have detected the virus. In support of this, we did not detect EBV in donor lymphocytes, but we were able to show, using STR profiling, that the xenografts were human and matched their corresponding donor lymphocytes. The lymphomas that developed in the initial transplantation were predominately from a B-cell lineage. However, two of the EBV^+^ lines had neither B nor T cell rearrangements, yet were CD45^+^. Within the classification of non-B lineage lymphoblastic lymphomas, 30% are divided into T-cell/NK bipotential progenitors, early stage T-cell precursors without TCR rearrangements, and NK precursors [30]. It is possible that both lines are derived from an early stage T-cell precursor. However, sequencing will be required to elucidate their origin.

The development of EBV-associated lymphoma from human solid tumour xenografts is not a new phenomenon, but it is under reported. It has been described in urothelial cancer [31], non-small cell lung cancers [32], hepatocellular carcinomas [33] and in prostate cancer [24]. The frequency of lymphoma development ranged from 17% (in SCID mice) to 80% in NSG/NOG mice. We observed a frequency of 82% in Rag2^-/-^γC^-/-^ mice, suggesting that the most severe immune deficient mouse models are more vulnerable to development of EBV-driven lymphoma, presumably due to the absence of cytotoxic T cells which play a critical role in the control of latent EBV infected B-cells [34].

The generation of serially-transplantable prostate cancer xenograft lines from primary specimens has rarely been reported. Of the few successful studies most have been derived from advanced metastatic specimens [35–39]. More recently Lin et al. [12] reported the establishment of five xenografts from primary specimens, in NOD/SCID mice, with a success rate of 27%. They attributed the improved survival rate to grafting the specimen under the renal capsule but did not carry out a comparison with other sites [12]. Nonetheless, it is likely that if the donor is EBV seropositive, engraftment under the renal capsule site is unlikely to prevent development of lymphoma. It has been suggested that this phenomenon might be avoided through the use of implantable slow-release testosterone pellets [37]. However, we were unable to prevent lymphoma development despite the use of androgen supplementation.

## Conclusion

Taken together, these data highlight the importance of thorough characterisation of xenograft outgrowths. We advocate early screening for EBV together with regular genotyping and phenotyping for lymphoid and epithelial markers, to avoid lymphoma development from donor lymphocytes and overgrowth of stable PDX by murine cells. Whilst we were unable to associate specific clinical characteristic with engraftment, due to the small numbers of stable xenografts derived from this program, we were able to show that prostate tumours have significantly longer latencies than lymphomas. Prostate cancer in humans is slow growing and it appears that this is mirrored in the mouse, particularly for the least aggressive tumours.

## Supporting information

S1 Fig Primary tumour xenografts derived from treatment-naïve human prostate tissue specimens. **A.** H&E sections of representative prostate cancer xenografts and their corresponding human donor sample… **B**. Xenograft tissue sections stained with antibodies raised against human AR (clone 441 at 1:50), PSA (1:25) and pan-cytokeratin (1:800). Human tissue, from patients with BPH or cancer was used as a positive control. Non-specific signal was assessed using isotype controls and secondary only antibodies. Xenograft images are from primary outgrowths (annotated as F1). Magnification x400.
(TIF)

S2 Fig **Androgen sensitivity of xenograft lines derived from hormone naïve donors: A.** Quantitative RT-PCR for flAR, variants AR-V1 and AR-V7. The results are expressed as normalised values (to GAPDH and a calibrator (LNCaP for AR-V7) or PC3 for flAR, and AR-V1. Each sample was run in triplicate and error bars represent mean ± SD of technical replicates. Unpaired, two-tailed T-tests were run to determine differences between cell lines and PDXs. ***P<0.001. **B**. Response of PDX lines to placebo (open bars) and the anti-androgen, flutamide (closed bars). Tumour response was calculated from the slope of log_10_ transformed tumour growth curves. *** P<0.0001, unpaired t-test. **B. C**.
(TIF)

S1 Table PDX (between P1-P10) were validated as unique to the patient donor by short tandem repeat (STR) DNA fingerprinting using the Promega Powerplex 16 system. The STR profiles were matched to their respective lymphocyte DNA.–indicates loss of specific loci.
(DOCX)

S2 Table Single nucleotide variants (SNVs) and mutations for PDX H455. (XLSX)

S3 Table Verification of human IgH gene rearrangements from PCR amplicons. Results of Blast search from Sanger sequences.
(DOCX)
