# Supplementary figures and images for: Spontaneous development of Epstein-Barr Virus associated human lymphomas in a prostate cancer xenograft program
Source: PLoS One. 2017 Nov 16;12(11):e0188228. doi: 10.1371/journal.pone.0188228 (PMC5690647; doi:10.1371/journal.pone.0188228)

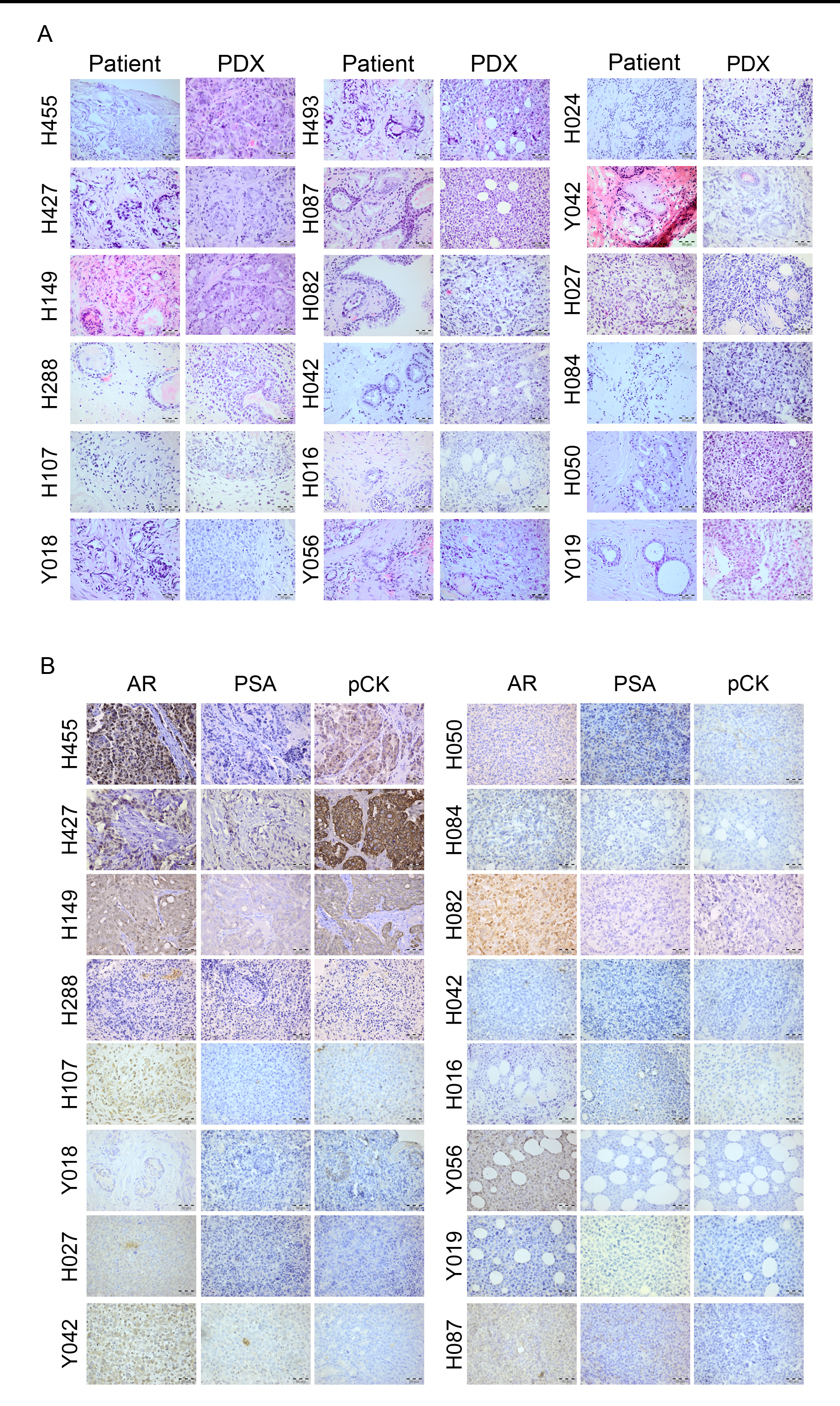

Supplement: S1 Fig — A. H&E sections of representative prostate cancer xenografts and their corresponding human donor sample… B. Xenograft tissue sections stained with antibodies raised against human AR (clone 441 at 1:50), PSA (1:25) and pan-cytokeratin (1:800). Human tissue, from patients with BPH or cancer was used as a positive control. Non-specific signal was assessed using isotype controls and secondary only antibodies. Xenograft images are from primary outgrowths (annotated as F1). Magnification x400. (TIF) [file pone.0188228.s001.tif]

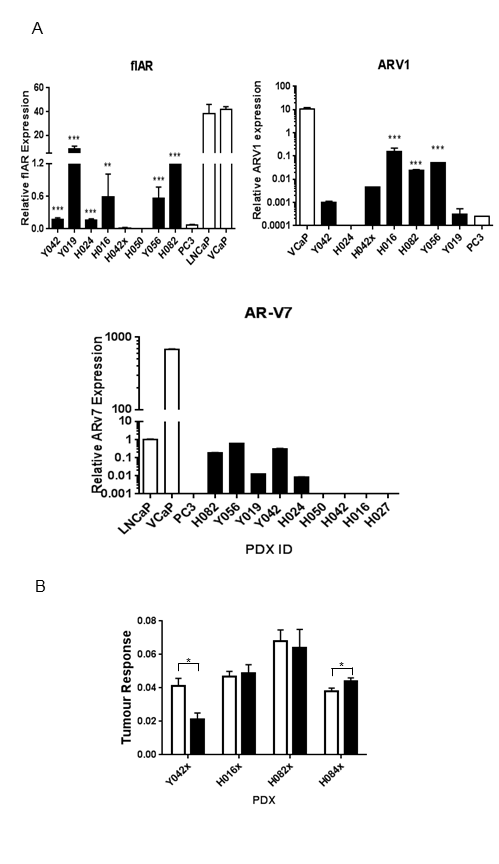

Supplement: S2 Fig — Androgen sensitivity of xenograft lines derived from hormone naïve donors: A. Quantitative RT-PCR for flAR, variants AR-V1 and AR-V7. The results are expressed as normalised values (to GAPDH and a calibrator (LNCaP for AR-V7) or PC3 for flAR, and AR-V1. Each sample was run in triplicate and error bars represent mean ± SD of technical replicates. Unpaired, two-tailed T-tests were run to determine differences between cell lines and PDXs. ***P<0.001. B. Response of PDX lines to placebo (open bars) and the anti-androgen, flutamide (closed bars). Tumour response was calculated from the slope of log10 transformed tumour growth curves. *** P<0.0001, unpaired t-test. B. C. (TIF) [file pone.0188228.s002.tif]
