# Supplementary material for: Spontaneous development of Epstein-Barr Virus associated human lymphomas in a prostate cancer xenograft program
Source: PLoS One. 2017 Nov 16;12(11):e0188228. doi: 10.1371/journal.pone.0188228 (PMC5690647; doi:10.1371/journal.pone.0188228)
Supplement: S1 Table — The STR profiles were matched to their respective lymphocyte DNA.–indicates loss of specific loci. (DOCX) [file pone.0188228.s003.docx]

| **PDX** | **Locus** | | | | | | | | | | | | | | | |
| --- | --- | --- | --- | --- | --- | --- | --- | --- | --- | --- | --- | --- | --- | --- | --- | --- |
|  | **AMEL** | **CSF1PO** | **D13S317** | **D16S539** | **D18S51** | **D21S11** | **D3S1358** | **D5S818** | **D7S820** | **D8S1179** | **FGA** | **PentaD** | **PentaE** | **TH01** | **TPOX** | **vWA** |
| Y042 | XY | 10,12 | 10,12 | 11,- | 16,17 | 28,30.2 | 15,17 | 12,12 | 10,10 | 12,14 | 20,22 | 13,13 | 10,16 | 6,7 | 8,12 | 14,17 |
| H016 | XY | 11,12 | 11,11 | 8,13 | 17,18 | 30,30 | 16,17 | 12,12 | 9,11 | 13,15 | 22,22 | 9,12 | 17,18 | 6,9.3 | 8,11 | 16,17 |
| H024 | XY | 10,13 | 8,11 | 10,11 | 11,14 | 29,31,2 | 14,15 | 12,12 | 8,8 | 10,15 | 19,22 | 9,10 | 10,19 | 6,9.3 | 8,8 | 17,18 |
| H042 | XY | 10,10 | 8,9 | 11,13 | 15,18 | 30,32,2 | 17,17 | 11,12 | 8,12 | 12,14 | 22,25 | 10,11 | 7,12 | 6,7 | 9,12 | 17,18 |
| H084 | XY | 10,12 | 12,12 | 9,10 | 12,15 | 29,30 | 15,16 | 11,12 | 9,10 | 13,14 | 19,22 | 11,13 | 10,14 | 8,9.3 | 11,12 | 16,18 |
| H082 | XY | 11,12 | 11,12 | 12,13 | 12,18 | 29,30 | 14,14 | 12,13 | 9,11 | 12,13 | 21,26 | 8,12 | 11,15 | 6.6 | 11,12 | 16,18 |
| H087 | XY | 11,13 | 11,12 | 9,12 | 13,15 | 30,32 | 15,16 | 11,11 | 10,11 | 11,11 | 23,24 | 11,13 | 11,13 | 6,9.3 | 10,11 | 14,16 |
| H050 | XY | 10,12 | 12,13 | 9,11 | 15,18 | 28,31 | 18,18 | 11,12 | 8,10 | 11,13 | 22,25 | 12,13 | 12,15 | 8,9.3 | 11,11 | 16,18 |
| H288 | XY | 11,12 | 11,11 | 12,14 | 12,16 | 27,30 | 14,17 | 11,12 | 8,10 | 12,14 | 18,20 | 9,12 | 12,15 | 6,6 | 11,11 | 17,18 |
| H070 | XY | 9,10 | 11,13 | 11,11 | 15,18 | 28,32,2 | 16,17 | 11,11 | 8,10 | 8,15 | 20,23 | 12,13 | 12,14 | 6,9.3 | 8,11 | 15,16 |
| H027 | XY | 12,12 | 10,12 | 12,12 | 13,18 | 27,29 | 16,16 | 11,12 | 10,10 | 13,15 | 22,23 | 10,12 | 11,13 | 7,8 | 8,11 | 14,14 |
| H107 | XY | 12,12 | 10,12 | 13,13 | 12,16 | 27,28 | 16,16 | 13,14 | 9,12 | 13,14 | 20,20 | 9,14 | 13,13 | 9.3,9.3 | 8,11 | 14,18 |
| H427 | X- | 10,13 | -,13 | 9,12 | 12,19 | -,31,2 | 15,15 | 11,12 | 10,13 | 12,14 | 20,22 | 11,12 | 7,11 | 6,9.3 | 8,8 | -,18 |
| H460 | XY | 11,12 | 10,12 | 10,13 | 14,15 | 29,29 | 16,17 | 11,13 | 10,13 | 13,13 | 23,24 | 9,12 | 17,17 | 6,7 | 8,8 | 15,18 |
| H493 | XY | 11,12 | 12,12 | 9,11 | 12,14 | 28,33,2 | 15,16 | 12,12 | 9,10 | 12,15 | 20,24 | 11,13 | 13,13 | 6,8 | 8,9 | 15,20 |
| Y019 | XY | 11,12 | 11,11 | 12,13 | 12,21 | 30,30 | 15,15 | 11,12 | 9,12 | 10,13 | 24,24 | 9,10 | 11,18 | 6,9.3 | 8,11 | 17,- |
| Y018 | XY | 11,11 | 9,11 | 11,12 | 16,16 | 28,28 | 17,17 | 12,13 | 11,12 | 10,14 | 23,24 | 12,13 | 11,14 | 5,9.3 | 8,8 | 16,17 |
| Y056 | XY | 12,13 | 11,12 | 12,13 | 12,18,19 | 30,33 | 16,17 | 11,12 | 9,11 | 14,14 | 22,23 | 9,10 | 7,17 | 9.3,9.3 | 9,11 | 14,16 |
| H149 | XY | 10,13 | 9,11 | 11,13 | 10,14 | 28,29 | 15,17 | 12,12 | 8,8 | 12,13 | 20,21 | 13,13 | 5,14 | 9.3,9.3 | 8,11 | 19,19 |
| H455 | X- | 10,12 | 12,12 | -,12 | **-**.19 | 31,31.2 | 14,18 | 9,12 | 9,11 | 12,13 | 19,23 | 9,13 | 5,7 | 7,9.3 | 8,12 | 16,17 |
