# Supplementary material for: Spontaneous development of Epstein-Barr Virus associated human lymphomas in a prostate cancer xenograft program
Source: PLoS One. 2017 Nov 16;12(11):e0188228. doi: 10.1371/journal.pone.0188228 (PMC5690647; doi:10.1371/journal.pone.0188228)
Supplement: S3 Table — Results of Blast search from Sanger sequences. (DOCX) [file pone.0188228.s005.docx]

| **Sample ID** | **Blast search** | | | |
| --- | --- | --- | --- | --- |
|  | **Target** | **FR-JH** | **FR3-JH** | **DH-JH** |
| B-cell lymphoma |  | IgHV-J | IgHV-J | IgHD-J |
| H042 |  | IgHV-J | - | - |
| H460 |  | - | IgHV-J | - |
| Y018 |  | - | No significant similarity found | - |
| H460 |  | - | - | IgHD-J |
| H493 |  | - | - | IgHD-J |
| Y019 |  | - | - | IgHD-J |
| Y056 |  | - | - | IgHD-J |
